# Liver Transplant in a Patient under Methylphenidate Therapy: A Case Report and Review of the Literature

**DOI:** 10.1155/2015/437298

**Published:** 2015-01-22

**Authors:** Hoi Y. Tong, Carmen Díaz, Elena Collantes, Nicolás Medrano, Alberto M. Borobia, Paloma Jara, Elena Ramírez

**Affiliations:** ^1^Department of Clinical Pharmacology, Hospital Universitario La Paz, IdiPaz, School of Medicina, Universidad Autónoma de Madrid, Paseo de la Castellana 261, 28046 Madrid, Spain; ^2^Pediatric Hepatology Department, Hospital Universitario La Paz, IdiPaz, Paseo de la Castellana 261, 28046 Madrid, Spain; ^3^Pathological Anatomy Department, Hospital Universitario La Paz, IdiPaz, Paseo de la Castellana 261, 28046 Madrid, Spain

## Abstract

*Background*. Methylphenidate (MPH) is widely used in treating children with attention-deficit-hyperactivity disorder. Hepatotoxicity is a rare phenomenon; only few cases are described with no liver failure. *Case*. We report on the case of a 12-year-old boy who received MPH for attention-deficit-hyperactivity disorder. Two months later the patient presented with signs and symptoms of hepatitis and MPH was discontinued, showing progressive worsening and developing liver failure and a liver transplantation was required. Other causes of liver failure were ruled out and the liver biopsy was suggestive of drug toxicity. *Discussion*. One rare adverse reaction of MPH is hepatotoxicity. The review of the literature shows few cases of liver injury attributed to MPH; all of them recovered after withdrawing the treatment. The probable mechanism of liver injury was MPH direct toxicity to hepatocytes. In order to establish the diagnosis of MPH-induced liver injury, we used CIOMS/RUCAM scale that led to an assessment of “possible” relationship. This report provides the first published case of acute MPH-induced liver failure with successful hepatic transplantation. *Conclusions*. It is important to know that hepatotoxicity can occur in patients with MPH treatment and monitoring the liver's function is highly recommended.

## 1. Introduction

Methylphenidate hydrochloride (MPH) is a chain substituted amphetamine derivative that primarily acts as norepinephrine-dopamine reuptake inhibitor. The Food and Drug Administration (FDA) first approved MPH on 1955; however, it was not until the 1990s when MPH saw a dramatic increase in its prescription. In the PATS study almost one-third of the children revealed some side effects, mainly weight loss and neurological effects [1]. A few scattered and sporadic cases of hepatotoxicity with MPH treatment have been reported and usually referred to transient elevation of liver enzymes. This report describes a case of irreversible methylphenidate-induced liver failure.

## 2. Case Presentation

**Figure 1:**
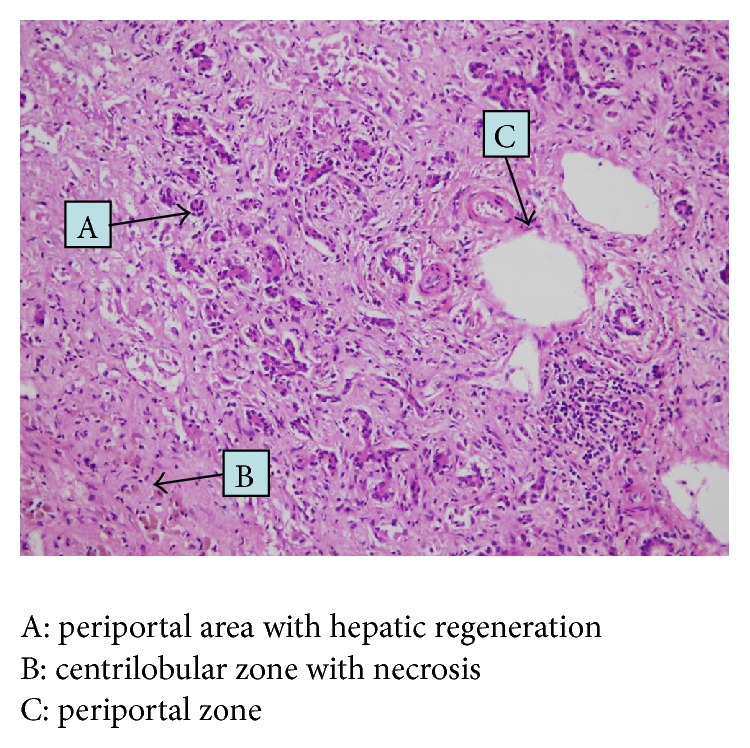
Liver biopsy.

A 12-year-old boy with no relevant medical history was treated with MPH at an appropriate dose of 30 mg daily for attention-deficit-hyperactivity disorder (ADHD), and no other treatment was received in the previous months. After two months of treatment, the patient presented with a 2-day history of generalized itching, malaise, fatigue, and anorexia and with no fever. At that time, MPH was discontinued. Initial aminotransferases (alanine aminotransferase, ALT; aspartate aminotransferase, AST), total bilirubin, and alkaline phosphatase were elevated, while hepatitis panel (HBsAg, anti-HBcore, anti-HAV, anti-HIV, CMV IgM, and syphilis) was negative, and the patient's health continued to worsen in the next two months and finally he developed signs of liver failure and was transferred to Spain for hepatic transplantation. When the patient arrived, his liver function continued to deteriorate, and laboratory test on the first day determined the following levels: ALT of 155 U/L, AST of 310 U/L, and total serum bilirubin of 28.7 mg/mL, coagulation disorders (prothrombin activity of 13% and international normalized ratio of 4.9). After two days, the patient developed encephalopathy, with hyperammonemia (178 *µ*cg/dL), he was translated to intensive care unit (Table 1). Alternative diagnoses were ruled out through immunological test (antinuclear antibodies, ANA; smooth muscle antibody; LKM antibody) negatives. Alpha-fetoprotein was negative. Infectious origin through microbiological test revealed the following: Enterovirus was negative; Herpes simplex virus IgM, negative; CMV IgG, positive; CMV IgM, negative; Epstein-barr VCA IgM, negative; anti-EBNA IgG, positive; Parvovirus IgM, negative; Parvovirus IgG, positive; IgM, negative; Adenovirus, negative; the hepatitis panel (HBsAg, anti-HB core, anti-HVA, anti-HVC, and anti-HVE), negative; anti-HIV, negative; Toxoplasma IgG, positive; Toxoplasma IgM, negative; and Syphilis, negative. Serum ceruloplasmin was 15.4 mg/dL (normal ranges 20–60 mg/dL) and serum copper was 68 mcg/dL (normal ranges 50–150 mcg/dL). Abdominal ultrasound revealed a decreased hepatic size, the caudate lobe was prominent, and there were images of periportal fibrosis, the bile duct was of normal caliber. On the 4th hospitalization day in Spain, successful liver transplantation was performed. Liver biopsy reported parenchyma showing conserved architecture with bridging perivenular submassive necrosis; periportal hepatocytes showed pseudoacinar change and cholangiolar reaction. In the best preserved areas, the hepatocytes had intrahepatic and canalicular cholestasis. The portal tract had normal morphology with no evidence of inflammatory or thrombotic phenomenon. At any level acute or chronic inflammatory infiltrates, abscesses, or eosinophils were not observed (Figure 1). Patient gradually improved over the next weeks and the liver function showed a normalization trend, and MPH has not been restarted and for the next 2 years the patient has been well controlled with no further hepatic alteration events.

## 3. Discussion

ADHD is a common neurobehavioral disorder and one of the most prevalent chronic health problems in childhood [1]. The current estimated prevalence of ADHD is 2–6% among preschool-age children and 3–7% for school-age children [2]. Recently, practice guidelines support the benefits of treatment with both behaviour therapy and MPH, which is the most commonly prescribed psychostimulant [3]. Common side effects of MPH include loss of appetite and anxiety, and the most worrying side effect was a small but significant impact on the cardiovascular system including increases in blood pressure and heart rate as well as sudden cardiac death [4, 5]. However, one known but rare adverse effect of MPH is hepatotoxicity. Only few case reports of liver injury attributed to MPH have been published, possibly due to the fact that most of the patients generally develop mild, asymptomatic, and reversible elevation of liver chemistries. The first case of hepatotoxicity due to MPH was described in 1972. In the case of a 67-year-old woman with MPH treatment, laboratory test showed elevated aminotransferases and alkaline phosphatase and MPH was discontinued and her liver's enzymes normalized [6].

The mechanism of hepatotoxicity associated with most drugs is idiosyncratic, which implies that drug-induced liver injury (DILI) develops in only a small proportion of subjects exposed to a drug in therapeutic doses, and must be consider the interaction between genetic and environmental risk factors making DILI unpredictable for most hepatotoxins. Thereby, we have found two case reports whose mechanism of hepatotoxicity of MPH could be idiosyncratic. They were patients with normal liver function previously. In one case after 5 weeks and in the other case after 3 months of onset of MPH therapy, elevated levels of aminotransferases and bilirubin were presented and alternative diagnostics were excluded. MPH was discontinued and liver's enzymes decreased [7, 8].

Allergy idiosyncratic hepatotoxicity is another possible mechanism of DILI, characterized by the presence of fever, skin reactions, eosinophilia, and formation of autoantibodies [9]. The other two cases in the literature can support this possible causal mechanism of MPH-induced hepatotoxicity. First, for the case of a 19-year-old black woman who had been injected intravenously with MPH and was admitted for jaundice, fever, and pain in the right upper abdomen, laboratory data showed elevated liver enzymes; a liver biopsy was performed revealing portal inflammation with lymphocytes, plasma cells, and eosinophils. Autoantibodies were not reported. Patient gradually got better the next 2 weeks and was given injection of MPH intravenously for two days after recovery and liver enzymes again showed a significant increase, proving positive rechallenge effect which strengthens the link of hepatotoxicity due to MPH [10]. The other case was reported by Lewis et al. a 57-year-old Caucasian male with a history of orthotopic liver transplantation 4 years before because of chronic hepatitis C, had maintained stable treatment and the liver's enzymes had been normal after transplantation. On routine laboratory evaluation that discovered elevation of ALT, AST, and bilirubin, the only new medication that began 1 month earlier was MPH for depressive symptoms. Immunologic tests reported positive ANA, positive anti-SMA, negatives antimitochondrial antibody and anti-LKM, and elevated serum IgG immunoglobulins. A liver biopsy showed severe lobular and periportal necroinflammatory infiltrate with predominance of lymphocytes, plasma cells, and eosinophils, consistent with autoimmune hepatitis. MPH therapy was discontinued and liver's enzymes returned to previous levels [11].

MPH is a drug whose toxicity is increased by adrenergic agonist drugs [12]. A study in mice proved that when MPH is given as a single dose of 75 to 100 mg/Kg, it produced hepatic necrosis in male mice and when coadministered with beta-2 adrenoreceptors drugs can produce important potentiation of the liver injury by the increase in the MPH concentration [13]. In the literature, the cardiovascular effects of the sympathomimetic amines (increase in the heart rate, blood pressure, and blood vessel contraction) [14] have been described as well as cases of ischemic events (myocardial infarction and stroke) and sudden death in children and adults taking ADHD stimulants [4, 15]. For this reason we cannot discard that the overall low flow of blood in the liver could be another mechanism of MPH-induced liver injury.

In our case, we think that the mechanism of liver injury was MPH direct toxicity to hepatocytes as an idiosyncratic reaction, and we cannot support that the liver failure was due to autoimmune hepatitis, because of the negative findings of immunological test (ANA, smooth muscle antibody, and LKM antibody) and the absence of inflammatory damage or infiltration by plasma cells, lymphocytes, or eosinophils in the explanted liver [16]. And we do not have data on ischemia hepatopathy. In order to establish the diagnosis of DILI [17] we used CIOMS/RUCAM scale [18] that led to the assessment of “possible” relationship.

All cases reported were mild and recovered after withdrawing MPH, but in contrast, the case of our patient was severe and he was referred for liver transplantation. Our review of possible MPH-induced liver injury indicates a spectrum of presumed hepatotoxicity ranging from mild elevation of aminotransferases with spontaneous recovery after withdrawal of MPH to severe fulminant hepatitis requiring liver transplantation.

In conclusion, drug-induced liver injury (DILI) represents a frequently adverse drug reaction. Drugs account for 20–40% of all instances of fulminate hepatic failure. Approximately 75% of the idiosyncratic drug reactions result in liver transplantation or death [19]. It is important to know that although rarely but subacute liver failure can occur in patients with MPH treatment and must be taken into account by clinicians. This is the first case report of liver transplantation due to MPH therapy. This case has been reported to the National Pharmacovigilance Agency of Spain (registered as number 3433).

## Figures and Tables

**(a):** 

Date	Episode	ALT (normal, <35) UI/L	AST (normal, <45) UI/L	Total bilirubin (normal, 0–1.2) mg/dL	Alkaline phosphatase (normal, 30–355) UI/L	Prothrombin activity (80–120) %
18/12/10	Control	13	21	0.3	56	101
26/02/11	Jaundice, *Coluria*, acholia MPH was discontinued	423	857	4	339	71
04/04/11	Worsening coagulopathy	182	361	12.2	304	36

**(b):** 

Date	Episode	ALT (normal, 30–65) UI/L	AST (normal, 15–37) UI/L	GGT (normal, 5–85) UI/L	Total bilirubin (normal, 0.2–1.4) mg/dL	Alkaline phosphatase (normal, 42–362) UI/L	Prothrombin activity (80–120) %
04/05/11	Arrived to Hospital Universitario La Paz	138	310	29	28.7		13
05/05/11	Onset of NAC	141	332	21	36.9	275	17
06/05/11	Encephalopathy medium-severe intensity with hyperammonemia	122	269	29	27		27
07/05/11		119	238	29	27.4		19
08/05/11	Hepatic transplantation	110	243	30	26.9		21
08/05/11	After hepatic transplantation	480	792	44	10.8		41
09/05/11		534	996	48	6.9		51
10/05/11		389	373	43	3.9		97
11/05/11		348	213	135	5.4		105
12/05/11		356	185		5.4		94
13/05/11		310	124	494	5.6		102
14/05/11		259	78	511	4.7		108
15/05/11		269	104	737	4.9		109
16/05/11		260	93	703	4.1		118
17/05/11		377	193	1106	4.7		109
18/05/11		459	194	1099	4.4		113
19/05/11		478	188	1139	3.9		107
20/05/11	Discharge from ICU	338	86	946	3		115
21/05/11		279	64	939	2.7		105
22/05/11		206	39	782	2.4		104
23/05/11		165	30	745	2.3	260	99
24/05/11		127	25	629	2		95
26/05/11		111	35	604	1.8		103
28/05/11		93	33	522	1.6		97
31/05/11		75	36	417	1.4		108
03/06/11		82	39	351	1.2	192	119
07/06/11	Discharge from the hospital	61	22	305	1.3	189	113
10/06/11		42	25	262	2.13	220	105
20/06/11		23	20	152	1.14	188	104

NAC: N-acetylcysteine.

## Abbreviations

ADHD: Attention-deficit-hyperactivity disorder
ALT: Alanine aminotransferase
ANA: Antinuclear antibody
Anti-HBc: Hepatitis B core antibody
Anti-HBs: Hepatitis B surface antibody
Anti-SMA: Anti-smooth muscle antibody
Anti-VHA: Hepatitis A antibody
Anti-VHB: Hepatitis C antibody
Anti-VHE: Hepatitis E antibody
AST: Aspartate aminotransferase
CMV: Cytomegalovirus
DILI: Drug-induced liver injury
FDA: U.S. Food and Drug Administration
LKM: Liver-kidney microsome antibodies
MPH: Methylphenidate hydrochloride.
